# Evaluating an Advance Care Planning Skills Training for Medical, Nursing, and Social Work Students

**DOI:** 10.1093/geroni/igaa057.784

**Published:** 2020-12-16

**Authors:** Todd Becker, John Cagle

**Affiliations:** University of Maryland, Baltimore, Baltimore, Maryland, United States

## Abstract

Advance care planning (ACP) helps to ensure future healthcare is congruent with individual preferences. Curricula for health-focused professions rarely include ACP content. This is a repeated measures evaluation of an innovative, interprofessional student training to enhance the ability to lead ACP discussions. Outcomes were measured via self-report surveys at two timepoints (baseline = T1 [n = 35]; posttraining follow-up = T2 [n = 20]) and included validated measures of communication self-efficacy, ACP self-efficacy, and interprofessional teamwork. Data collection from a third timepoint (T3), following randomized group assignment is currently underway. The matched T1–T2 sample (N = 17) included students from medicine (64.7%), nursing (17.6%), and social work (17.6%). Participants were largely young (M=26 years), non-Hispanic (94.0%), White (59.0%), and female (71.0%). Paired t tests examined change from T1 to T2 for all outcomes. Despite worse communication self-efficacy (Mdiff=1.6, p<.001) and ACP self-efficacy (Mdiff=.92, p<.001), perceptions of interprofessional teamwork improved (Mdiff=3.0, p=.008). These T1–T2 findings mirror results from similar, prior research, which subsequently discovered that both self-efficacy outcomes and their effect sizes for change worsened at T2, but ultimately and substantially improved at T3. These fluctuations suggest participants initially overestimated their self-efficacy related to ACP at T1 and corrected their appraisals at T2. Other studies should account for this self-correction. Further replication is needed to understand the dip that appears to occur before anticipated improvements occur.

